# Expanding the Frontiers of Tuberculosis Prevention: Moving from Targeted Preventive Therapy to Integrated Public Health Strategies

**DOI:** 10.3390/tropicalmed11080214

**Published:** 2026-07-29

**Authors:** Kannamkottapilly Chandrasekharan Prajitha, Anam Anil Alwani, Pruthu Thekkur

**Affiliations:** Centre for Operational Research, International Union Against Tuberculosis and Lung Disease, 75001 Paris, France; prajitha.kc.consultant@theunion.org (K.C.P.); anam.alwani.consultant@theunion.org (A.A.A.)

**Keywords:** tuberculosis infection, multisectoral accountability framework, End TB Strategy, sustainable development goals, operational research

## Abstract

Tuberculosis (TB) remains a leading cause of infectious disease-related mortality worldwide despite major advances in diagnostics, treatment, and programmatic control. Achieving the goals of the World Health Organization End TB Strategy requires a greater emphasis on prevention alongside improvements in diagnosis and treatment. This viewpoint examines evolving priorities in TB prevention and highlights key insights from studies included in the Special Issue ‘New Perspectives in Tuberculosis Prevention and Control’. The articles published in the Special Issue highlight the importance of identifying populations at increased risk of infection, disease transmission, and disease progression; strengthening the implementation of tuberculosis preventive treatment among household contacts and people living with HIV; and addressing the behavioral, social, and structural determinants that influence access to TB prevention and care. They also demonstrate persistent challenges related to medicine availability, healthcare worker capacity, patient initiation and follow-up, program monitoring, and the implementation of preventive interventions in resource-constrained settings. Emerging developments in diagnostics, biomarkers, vaccines, and digital health may contribute to the longer-term TB prevention agenda but require further evaluation of their feasibility, affordability, and programmatic relevance. Collectively, the evidence indicates that effective TB prevention extends beyond individual biomedical interventions and requires coordinated approaches involving healthcare systems, communities, surveillance systems, and social support mechanisms. Continued investment in implementation research, health-system strengthening, program management, economic evaluation, and multisectoral action will be critical to translate existing evidence into sustainable, equitable, and context-specific strategies for reducing the global burden of TB.

## 1. Background

Tuberculosis (TB) is the leading infectious cause of mortality globally despite decades of advances in diagnostics, treatment, and programmatic control strategies, including systematic case detection, standardized treatment, and public health interventions for TB control. The burden of disease continues to be concentrated in countries across South-East Asia, Western Pacific and African regions, which together account for 85% of global TB cases [1]. Historically, TB control programs largely focused on identifying and treating individuals with active disease, an approach that contributed substantially to reductions in mortality, but had limited impact on transmission dynamics at the population level [2]. In recent years, to achieve the ambitious goals of the World Health Organization End TB Strategy, including a 90% reduction in TB incidence and a 95% reduction in TB mortality by 2035, the global TB response has shifted toward strategies that emphasize not only improved diagnosis and treatment but also the prevention of disease [3].

The recent literature has highlighted several evolving areas within TB prevention, including improved diagnostics for the detection of tuberculosis infection (TBI), optimization of contact investigation strategies, expansion of tuberculosis preventive therapy (TPT), biomarker-guided risk stratification, vaccine development, and integrated public health approaches [2,4,5,6]. At the same time, important questions remain regarding implementation feasibility, the prioritization of target populations, health-system readiness, affordability, scalability, and the long-term sustainability of prevention interventions across different epidemiological settings [5,6,7].

The studies included in this Special Issue collectively illustrate the expanding scope of TB prevention and the operational realities associated with implementation in routine program settings. Together, they highlight three interconnected areas that increasingly shape prevention strategies: the identification of infection and transmission risk; the implementation of preventive therapy among high-risk populations; and the behavioral, social, and structural determinants that influence TB prevention, particularly access to prevention and care. These studies also demonstrate that TB prevention increasingly extends beyond a single biomedical intervention and instead requires coordinated approaches involving healthcare systems, communities, surveillance systems, and social support mechanisms. The interventions discussed in this viewpoint largely focus on secondary prevention strategies aimed at identifying tuberculosis infection, preventing progression to active disease, and interrupting onward transmission among populations at increased risk.

Accordingly, this viewpoint does not aim to provide a comprehensive review of all TB prevention strategies. Instead, it uses the studies published in this Special Issue as a lens to examine how the field of TB prevention is evolving. While many of the individual interventions discussed are well established, their collective contribution highlights an important shift in emphasis, from viewing prevention as a set of isolated biomedical interventions to recognizing it as an integrated public health approach that requires coordinated action across healthcare systems, communities, surveillance systems and social support mechanisms. This broader perspective forms the central viewpoint of this article.

## 2. Identifying Infection and Transmission Risk

A major challenge for TB control remains the large global reservoir of people with TBI. Recent estimates suggest that approximately one quarter of the global population may harbor TBI with Mycobacterium tuberculosis, although the risk of progression to disease varies considerably across populations and epidemiological contexts [2,8]. Preventive interventions therefore depend heavily on identifying individuals and populations at increased risk of infection, transmission, or progression to active disease [9].

Healthcare workers (HCWs) remain an important occupational risk group for TB exposure in high-burden settings. In a prospective cohort study conducted among HCWs in tertiary hospitals in India, Subramanian et al. reported a TBI prevalence of nearly 38%, with measurable rates of both conversion and reversion over time, suggesting ongoing occupational exposure within healthcare environments [10]. These findings are consistent with broader discussions regarding healthcare-associated TB transmission, and reinforce the continuing importance of infection prevention and control (IPC) measures in healthcare settings [11]. Recent reviews have highlighted the role of environmental control measures, respiratory protection, administrative interventions, and facility-level IPC strategies in reducing transmission risk, although implementation feasibility often varies according to healthcare infrastructure and resource availability [12,13].

Identification of TBI remains central to many prevention strategies. Conventional approaches such as the tuberculin skin test (TST) and interferon-gamma release assays (IGRAs) continue to be widely used, although both approaches have operational and interpretational limitations, particularly in Bacillus Calmette–Guérin (BCG)-vaccinated populations [2,5]. In addition, although TST and IGRAs are useful for identifying tuberculosis infection, neither test reliably predicts which individuals will progress to active disease, limiting their utility for individual risk stratification and reinforcing the need for improved predictive biomarkers [2,14]. Newer TB-specific antigen-based skin tests have emerged as potential alternatives, offering improved specificity and operational feasibility in some settings. However, programmatic factors such as affordability, cold-chain requirements, the need for repeat visits, and integration into existing guidelines may substantially influence the feasibility and utility of these newer diagnostic approaches in routine settings [7,15].Furthermore, additional evidence is needed to determine their long-term programmatic performance, cost-effectiveness, and role within existing diagnostic algorithms across different epidemiological settings. As shown in this Special Issue, Sathar et al. evaluated a TB antigen-based skin test in South Africa and highlighted practical considerations associated with its implementation in high-burden settings [16].

Beyond diagnostic innovations, long-term epidemiological surveillance remains important for understanding changing transmission dynamics and treatment outcomes across regions. A nationwide registry-based analysis of TB cases in Kazakhstan by Ryskulov et al. reported a decline in TB incidence rates and improvements in treatment outcomes over a ten-year period, while also identifying persistent disparities among specific demographic groups, including older individuals and men [17]. Such findings illustrate how surveillance data can support the identification of high-risk populations and inform context-specific prevention strategies.

## 3. Scaling Preventive Therapy Among High-Risk Populations

TPT has increasingly become a central component of global TB prevention strategies [1,4]. Several recent systematic reviews and meta-analyses continue to support the effectiveness of preventive therapy among high-risk populations, including people living with HIV (PLHIV) and household contacts [18,19,20]. Recent modeling analyses have also suggested that scaling short-course TPT among household contacts and PLHIV may provide substantial population-level impact while remaining cost-effective across high-incidence settings [21]. However, implementation of TPT remains variable across settings because of operational, logistical, and health-system constraints [22].

Current WHO guidelines recommend several short-course TPT regimens, including 3HP (once-weekly isoniazid plus rifapentine for 3 months), 1HP (daily isoniazid plus rifapentine for 1 month), 3HR (daily isoniazid plus rifampicin for 3 months), and 4R (daily rifampicin for 4 months), depending on the target population and local programmatic considerations [9].These shorter regimens have demonstrated improved treatment completion and acceptability compared with longer regimens. However, their successful implementation remains influenced by factors such as drug availability, procurement systems, healthcare worker training, and program readiness, particularly in resource-constrained settings [23,24].

PLHIV represent one of the populations at highest risk of progression from TBI to active disease. As shown in this Special Issue, Timire et al. evaluated TPT uptake among PLHIV receiving care in Zimbabwe and reported high treatment initiation following health-system strengthening interventions that included healthcare worker training and improved drug availability [25]. Similarly, Mande et al., in a mixed-methods study from Uganda, examined barriers and facilitators influencing TPT initiation and completion among PLHIV [26]. Although treatment completion among individuals initiating therapy was high, the study identified several operational barriers affecting implementation, including medication stock-outs (temporary shortages of TPT medicines), documentation gaps, and limitations in provider knowledge. Together, these studies highlight that successful implementation may depend not only on the availability of preventive regimens but also on broader health-system capacity and the integration of TB and HIV services.

These implementation barriers often reflect broader health-system constraints rather than limitations of preventive therapy itself. In many high TB burden settings, persistent challenges such as constrained healthcare resources, supply-chain disruptions, workforce shortages, and competing program priorities can hinder consistent delivery of TPT. In contrast, lower-burden settings may have different implementation priorities, with greater emphasis on identifying and reaching smaller high-risk populations through targeted approaches. Strengthening health systems, integrating TB prevention within routine healthcare services, and adopting context-specific implementation strategies will therefore be essential to improve program delivery across diverse epidemiological settings.

Household contacts of individuals with pulmonary TB continue to represent a priority population for preventive interventions [27,28]. The study from Zimbabwe by Mapuranga et al. examined household contact management and identified substantial attrition across the prevention cascade [29]. Although active case finding and contact follow-up were implemented systematically, major losses occurred during TPT eligibility assessment and treatment initiation stages, partly because of intermittent stock-outs of preventive therapy medicines. Encouragingly, treatment completion among those initiating therapy was high. These findings are consistent with the broader implementation literature demonstrating that losses frequently occur at multiple stages of the prevention cascade, including contact identification, screening, linkage to evaluation, and treatment initiation [5,28]. To overcome these challenges, recent studies have examined strategies intended to improve contact management and strengthen linkage to preventive therapy [30,31]. Implementation of timeliness metrics for household contact tracing and TPT delivery within public-sector settings has been explored using mixed-methods approaches involving TB champions [30]. The findings suggest that implementation-oriented approaches may improve coordination between contact identification and preventive therapy delivery within routine program settings. Nevertheless, successful scale-up of TPT continues to depend on sustained medicine availability, timely identification of eligible individuals, healthcare worker capacity, and program monitoring, which remain important implementation challenges in many high-burden settings.

Collectively, the studies included in this Special Issue demonstrate that the applicability of TB prevention strategies varies across epidemiological settings, reflecting differences in TB burden, health-system capacity, population risk profiles, and available resources. Consequently, successful implementation will require adaptation of these evidence-based prevention strategies to local epidemiological and programmatic contexts rather than following a uniform approach across settings.

## 4. Behavioral, Social and Structural Determinants of TB Prevention

Biomedical interventions alone are unlikely to eliminate TB in the absence of broader efforts addressing behavioral, social, and structural determinants of disease [32,33]. Factors including poverty, undernutrition, overcrowded housing, diabetes, smoking, migration, and limited healthcare access continue to influence both exposure risk and vulnerability to disease progression [34,35].

As shown in this Special Issue, a mixed-methods study conducted in South Africa by Sathar et al. explored patient experiences and barriers to TB care-seeking [36]. Participants described complex pathways before reaching formal healthcare services, including initial care-seeking from informal providers such as pharmacists and traditional healers. Financial barriers, transportation difficulties, work obligations, and stigma were frequently identified as factors contributing to delays in diagnosis and treatment. These findings are consistent with the broader literature demonstrating how social, behavioral and structural determinants may influence both access to healthcare and ongoing community transmission. Expanding TB prevention strategies to address such determinants through approaches including social protection programs and nutritional support may contribute to more sustainable reductions in TB incidence [32,33]. However, implementing these interventions remains challenging because many social and structural determinants extend beyond the health sector and require sustained multisectoral collaboration, long-term investment, and strong political commitment.

## 5. Future Priorities for TB Prevention

End-TB efforts over the past decade have significantly reduced TB incidence and mortality in high-burden countries. To achieve the goals outlined in the WHO End TB Strategy, we need to target the TB reservoir, the latent tuberculosis infection, given the lifelong risk of progression to active disease among high-risk populations. The studies included in the Special Issue highlight that the immediate priorities for TB prevention lie in strengthening the implementation of existing interventions within routine health systems. Although effective TPT regimens and programmatic guidelines are available, their delivery continues to be affected by medicine stock-outs, weaknesses in procurement and supply systems, documentation gaps, inadequate healthcare worker knowledge, and limitations in program capacity [25,26,29]. These findings indicate that future research should focus on identifying practical and context-specific approaches for integrating TPT within routine TB, HIV, and primary healthcare services, while ensuring sustained medicine availability, adequate provider training, and effective program monitoring [7,23,37].

Greater attention is also required across the entire cascade of contact investigation and preventive therapy. The studies from Uganda and Zimbabwe demonstrated that important losses may occur during contact identification, screening, eligibility assessment, and treatment initiation, even when treatment completion among individuals who initiate TPT is relatively high [26,29]. Future studies should therefore examine barriers at each stage of the prevention cascade rather than focusing only on treatment completion. Strengthening patient counseling, follow-up mechanisms, community-based service delivery, and linkage between contact investigation and TPT initiation may help reduce attrition and improve continuity of care [5,29,30,31].

Patient adherence and continued engagement with TPT program remain important challenges, particularly in high-burden settings. Adherence may be influenced by treatment duration, concerns regarding adverse events, limited understanding of the benefits of preventive therapy, competing work and family responsibilities, transportation difficulties, and the costs associated with repeated healthcare visits [26,36]. Future research should evaluate patient-centered approaches that simplify treatment delivery and reduce the burden of care. These may include decentralized treatment provision, integration of TPT visits with other healthcare services, community-based follow-up, and appropriately designed digital support mechanisms. However, such interventions should be assessed not only for their effectiveness but also for their acceptability, feasibility, equity, and sustainability within routine program settings.

Strengthening program implementation, patient follow-up, and program management will be essential for improving the population-level impact of TB prevention interventions. Routine monitoring systems should capture losses at each stage of the prevention cascade, including contact identification, screening, exclusion of active TB, assessment of eligibility, treatment initiation, adherence, completion, and management of adverse events [5,29,30]. Regular review of these indicators, supported by improved documentation, supportive supervision, and supply-chain monitoring, may allow national programs to identify implementation bottlenecks and undertake timely corrective action. Qualitative enquiry among patients, healthcare workers, and program managers may provide additional insights into the reasons for poor uptake, non-adherence, and loss to follow-up [26,29,37].

As countries expand TB prevention activities, particularly in high-burden, resource-constrained settings, greater consideration should be given to the affordability, cost-effectiveness, and budget impact of interventions. Strategies that are effective under research conditions may have limited programmatic value if they require expensive diagnostic procedures, repeated healthcare visits, specialized infrastructure, or substantial additional human resources. Economic evaluations should therefore examine not only the cost-effectiveness of individual TPT regimens but also the costs associated with contact investigation, eligibility assessment, treatment delivery, patient follow-up, and program management [4,9,21]. Such evidence will support national TB programs in selecting interventions that provide the greatest achievable population-level benefit within the resources available.

The findings of the Special Issue also reinforce the importance of addressing the social and structural determinants that influence access to TB prevention and care. Financial constraints, transportation difficulties, work obligations, stigma, and complex care-seeking pathways may contribute to delayed engagement with health services and poor continuity of care [36]. Policies aimed at reducing the financial burden of TB, improving living conditions, and expanding social protection programs may contribute to more equitable access to preventive interventions [32,33]. However, these efforts will require sustained multisectoral collaboration extending beyond the health sector, together with the meaningful involvement of affected communities and civil society [38].

Emerging areas such as more sensitive approaches for detecting subclinical TB, biomarker-guided risk stratification, omics-based research, and novel TB vaccines remain relevant to the longer-term prevention agenda [14,39,40,41]. Digital technologies may also have a role in supporting patient education, adherence, and follow-up [42]. However, these approaches will require further validation and careful assessment of their feasibility, affordability, cost-effectiveness, and potential for equitable implementation before widespread programmatic adoption.

These priorities represent different stages along the pathway from research to program implementation. Based on the evidence presented in this Special Issue, the immediate priority is to strengthen the implementation of existing TPT strategies, reduce losses across the prevention cascade, improve patient adherence and follow-up, and strengthen program monitoring and management. Emerging biomedical and technological approaches may complement these efforts in the future, but their contribution will depend on whether they can be translated into affordable, scalable, and sustainable interventions within high-burden health systems.

## 6. Conclusions

An estimated one-quarter of the world’s population is living with TBI, and subclinical tuberculosis is increasingly recognized as an important contributor of ongoing transmission-making stronger prevention efforts more critical than ever. The studies included in this Special Issue reflect the recognition that TB prevention operates across multiple levels, from identifying infection and expanding preventive therapy to addressing behavioral and structural determinants of transmission. Collectively, they highlight important implications for national TB programs and for implementing the WHO End TB Strategy through integrated, context-specific prevention policies. Advancing the global TB prevention agenda will require continued investment in research, health-system strengthening, multisectoral collaboration, and meaningful engagement of civil society, so that emerging evidence can be translated into sustainable, equitable, and context-specific strategies that reduce the global burden of tuberculosis.

## Data Availability

No new data were created or analyzed in this study. Data sharing is not applicable to this article.

## Abbreviations

The following abbreviations are used in this manuscript:

TB	Tuberculosis
TBI	Tuberculosis Infection
TPT	Tuberculosis Preventive Therapy
HCW	Healthcare Worker
IPC	Infection Prevention and Control
TST	Tuberculin Skin Test
IGRA	Interferon-Gamma Release Assay
BCG	Bacillus Calmette–Guérin
PLHIV	People Living with HIV
MDR TB	Multidrug-resistant Tuberculosis
